# Digitale Technologien zur Verbesserung der psychischen Gesundheit

**DOI:** 10.1007/s00103-024-03842-4

**Published:** 2024-01-31

**Authors:** Daniela C. Fuhr, Karin Wolf-Ostermann, Viktoria Hoel, Hajo Zeeb

**Affiliations:** 1 Abteilung für Evaluation und Prävention, Leibniz Institut für Präventionsforschung und Epidemiologie, Achterstr. 30, 28359 Bremen, Deutschland; 2 Gesundheitswissenschaften, Universität Bremen, Bremen, Deutschland; 3 Institut für Public Health und Pflegeforschung, Universität Bremen, Bremen, Deutschland

**Keywords:** Psychische Gesundheit, Digitalisierung, Deutschland, Mental health, Digitalization, Germany

## Abstract

Die Krankheitslast in Bezug auf psychische Erkrankungen ist enorm und wächst weltweit stetig. Der daraus resultierende steigende Bedarf an psychosozialer Hilfe schlägt sich auch in Deutschland negativ auf die Wartezeiten für Psychotherapieplätze nieder. Abhilfe können hier digitale Interventionen für die psychische Gesundheit schaffen, wie etwa Interventionen, die durch oder mithilfe einer Internetseite vermittelt werden (z. B. „Tele-Health“), Smartphone- oder Tablet-App-gestützte Interventionen sowie Interventionen, bei denen Textnachrichten oder virtuelle Realitäten Anwendung finden. In diesem Artikel wird zunächst eine Übersicht über die Funktionen und das Anwendungsspektrum von digitalen Technologien für die psychische Gesundheit gegeben. Die Evidenz von einzelnen digitalen Interventionsformen wird angesprochen. Insgesamt zeigt sich, dass im Vergleich zu keiner Therapie oder einer nicht-therapeutischen Kontrollgruppe digitale Interventionen für die psychische Gesundheit wahrscheinlich kosteneffizient sind. Neuere Ansätze wie das „digitale Phänotypisieren“ werden im Artikel erläutert. Abschließend werden einzelne Arbeiten aus dem „Leibniz WissenschaftsCampus Digital Public Health“ vorgestellt sowie Grenzen und Herausforderungen von Technologien für die psychische Gesundheit diskutiert.

## Abstract (en)

The burden of mental diseases is enormous and constantly growing worldwide. The resulting increase in demand for psychosocial help is also having a negative impact on waiting times for psychotherapy in Germany. Digital interventions for mental health, such as interventions delivered through or with the help of a website (e.g. “telehealth”), smartphone, or tablet app-based interventions and interventions that use text messages or virtual reality, can help. This article begins with an overview of the functions and range of applications of digital technologies for mental health. The evidence for individual digital forms of interventions is addressed. Overall, it is shown that digital interventions for mental health are likely to be cost-effective compared to no therapy or a non-therapeutic control group. Newer approaches such as “digital phenotyping” are explained in the article. Finally, individual papers from the “Leibniz ScienceCampus Digital Public Health” are presented, and limitations and challenges of technologies for mental health are discussed.

## Einleitung

Die weltweite Krankheitslast in Bezug auf psychische Erkrankungen ist enorm und wächst stetig. Im Jahr 2019 wurden ca. 970 Mio. Fälle von Forscher:innen der „Global Burden of Disease Study“ geschätzt, was einem Anstieg um fast 50 % in den letzten 30 Jahren entspricht [1]. Leider hat sich die weltweite psychische Belastung während der COVID-19-Pandemie noch weiter erhöht [2]. Die Kosten der mit psychischen Erkrankungen verbundenen Krankheitslast wurden im Jahr 2019 insgesamt weltweit auf ca. 5 Bio. US-Dollar geschätzt [3]. Psychische Erkrankungen beeinflussen das soziale Leben der Menschen negativ und sind deshalb weltweit als eine der Hauptursachen für Beeinträchtigungen und verminderte Lebensqualität anerkannt [1]. Die am häufigsten auftretenden psychischen Erkrankungen sind Depression, Angststörung und demenzielle Erkrankungen, die bei Frauen häufiger auftreten als bei Männern [1, 4, 5]. Andere psychische Erkrankungen, wie beispielsweise der Alkoholmissbrauch, finden sich hingegen häufiger bei Männern [4].

Die immer größer werdende Krankheitslast in Bezug auf psychische Erkrankungen und der daraus resultierende steigende Bedarf an psychosozialer Hilfe wirken sich negativ auf die Wartezeiten für Psychotherapieplätze aus, auch in Deutschland. Nach Angaben der Bundespsychotherapeutenkammer warteten im Jahre 2019 ca. 40 % der Patient:innen 3–9 Monate lang auf einen Psychotherapieplatz und es ist davon auszugehen, dass sich die Lage im Verlauf der Coronapandemie noch weiter verschärft hat [6]. Auf der anderen Seite sind psychische Erkrankungen weiterhin mit Stigma behaftet und ein Großteil der Bevölkerung sucht aufgrund von Scham und Angst vor Stigmatisierung keine oder zu spät Hilfe auf [7].

Einen ersten Zugang zu psychologischer Hilfe können auch digitale Technologien oder Gesundheitsanwendungen bieten und diese haben sich in den letzten beiden Jahrzenten rasant weiterentwickelt [8]. Digitale Interventionen für die psychische Gesundheit werden beispielsweise durch oder mithilfe einer Internetseite vermittelt (z. B. „Tele-Health“), sie können auch Smartphone- oder Tablet-App-gestützt sein oder es handelt sich um Interventionen, bei denen Textnachrichten oder virtuelle Realitäten eingesetzt werden [9].

Dieser Artikel zielt darauf ab, eine Gesamtübersicht über die Funktionen und das Anwendungsspektrum von digitalen Technologien in Bezug auf die Verbesserung der psychischen Gesundheit zu geben. Die Evidenz von einzelnen Interventionsformen wird angesprochen und neuere Ansätze wie die „digitale Phänotypisierung“ werden erläutert. Anschließend werden einzelne Arbeiten aus dem „Leibniz WissenschaftsCampus Digital Public Health“ (LWC DiPH) vorgestellt. Grenzen und Herausforderungen von Technologien für die psychische Gesundheit werden abschließend diskutiert.

## Anwendungsgebiete und Evidenz von digitalen Technologien im Bereich psychische Gesundheit

Digitale Technologien, die im Bereich der psychischen Gesundheit eingesetzt werden, haben ein breites Anwendungsspektrum von der Prävention über die Diagnose bis hin zur Behandlung [10]. Auch die Wissensvermittlung zur Aufklärung über psychische Symptome ist eine häufige Funktion digitaler Interventionen. Sie können spezifisch auf die Symptome einer psychischen Erkrankung, wie Depression, posttraumatische Belastungsstörung oder Demenz [11], oder auch transdiagnostisch ausgerichtet sein, wenn übergreifende Symptome über Störungsbilder hinaus behandelt werden (beispielsweise gleichzeitig Depression und Angststörung; [12]). Die Interventionsstrategien und Inhalte von digitalen Interventionen sind sehr divers und entsprechend sind auch Unterschiede in ihrer Effektivität zu beobachten [13]. Im Vergleich zu keiner Therapie oder einer nicht-therapeutischen Kontrollgruppe zeigt sich jedoch, dass digitale Interventionen für die psychische Gesundheit wahrscheinlich kosteneffizient sind [14]. Allerdings ist der Kosten-Nutzen-Unterschied zwischen digitalen Interventionen und traditionellen Formen der Psychotherapie noch unklar [14].

Apps sind eine der am häufigsten entwickelten Interventionsformen zur digitalen Behandlung von psychischen Problemen. Hier besteht insbesondere in Bezug auf Depression und Angststörung eine gute Forschungslage und Studien zeigen mittlere Effekte in Bezug auf eine Verbesserung der Symptomatik unter Verwendung von Methoden aus der kognitiven Verhaltenstherapie [15–18]. Bei posttraumatischen Belastungsstörungen und Schizophrenie sind die Ergebnisse eher unklar [19–21]. Neben Apps werden häufig Chatbots verwendet, die insbesondere im Rahmen der Diagnostik hilfreich sein können, indem sie Screeningfunktionen für Depression, Angststörungen oder Alkoholmissbrauch übernehmen. Chatbots können zudem für das Selbstmanagement der eigenen psychischen Gesundheit, für Beratung oder für eine Informationsübermittlung (bspw. in Form einer psychosozialen Edukation) eingesetzt werden [11].

Digitale Interventionen, insbesondere Apps, können entweder „geleitet“ sein (Unterstützung und Feedback durch Therapeut:in in Form von Textnachrichten oder Telefonanrufen) oder sie werden „ungeleitet“ durchgeführt. Die momentane Forschungslage legt nahe, dass geleitete digitale Interventionen effektiver sind als ungeleitete [22]. Grundsätzlich ist die Abbruchquote bei digitalen Interventionen hoch. Es gibt aber Hinweise, dass die Teilnahme in einem klinischen Setting besser ist und auch, wenn ein/e Therapeut:in an der Intervention beteiligt ist [23]. Strategien, um Patient:innen in einer digitalen Intervention für die psychische Gesundheit zu halten, wurden auch von Wissenschaftler:innen des Leibniz WissenschaftsCampus Digital Public Health (LWC DiPH) untersucht. Diese reichen von Feedback in Bezug auf das eigene Wohlergehen über die Beantwortung von Fragen zu Inhalten der Intervention bis hin zur Einbindung der Teilnehmer:innen in soziale Foren [22, 24].

Digitale evidenzbasierte Interventionen können bei bestimmten psychischen Erkrankungen und eher leichten psychischen Symptomen hilfreich sein. Es ist jedoch fraglich, ob digitale Interventionen eine Psychotherapie mit einer/m Therapeut:in komplett ersetzen können oder ob dies überhaupt wünschenswert ist. Eher angedacht werden neue hybride Therapieformen, in denen digitale Interventionen mit traditioneller Verhaltenstherapie verknüpft werden [12]. Dies ist auch in deutschen Behandlungsleitlinien so vorgesehen. Beispielsweise schlagen die „Nationale Versorgungsleitlinie zur Unipolaren Depression“ und die S3-Leitlinie „Psychosoziale Therapien bei schweren psychischen Erkrankungen“ bei mittelschwerer oder schwerer Depression eine digitale und evidenzbasierte Intervention als zusätzliche Interventionsform vor [25–27]. Als zusätzliche Interventionsform können digitale Interventionen auch außerhalb psychiatrischer oder psychotherapeutischer Behandlung von Hausärzt:innen verordnet werden. Studien in Deutschland zeigen, dass Patient:innen mit milden oder moderaten Symptomen einer Depression von einer Internetintervention profitieren, wenn diese zusätzlich zur Standardtherapie (bspw. Medikation oder Überweisung an eine/n Psychotherapeut:in) angeboten werden [28].

## Neuere Ansätze: digitale Phänotypisierung

In den letzten Jahren hat sich die digitale Phänotypisierung (Digital Phenotyping) als neuer Forschungsansatz in Bezug auf die psychische Gesundheit etabliert [29]. Hierbei geht es darum, Daten einer Person mittels ihres Smartphones oder ihrer Smartwatch zu sammeln und diese zur Diagnose, Vorhersage und Überwachung von Problemen bei der psychischen Gesundheit zu nutzen [30]. Mittels digitaler Phänotypisierung können 3 Arten von Daten gesammelt werden: sensorische Daten, Aktivitätsdaten und Daten in Bezug auf die sozialen Medien [30]. Sensorische Daten umfassen mehrheitlich GPS-Daten, die Informationen über den Aufenthaltsort des/der Patient:in und ihr Bewegungsmuster liefern. Aktivitätsdaten beziehen sich auf die Schlaf- und Wachzeiten der Person, die Anzahl der erhaltenen Text- oder Sprachnachrichten und die Verweildauer in sozialen Medien und im Internet. Daten in Bezug auf die sozialen Medien gehen einen Schritt weiter und analysieren die genutzten Inhalte sowie die Posts der Person (bspw. wird analysiert wann, wie und was gepostet wird; [30]). Diese „digitalen Biomarker“ können dann mittels Machine Learning analysiert werden, um beispielsweise eine Verbesserung oder einen Rückfall in eine Depression vorherzusagen [31]. Auch können bestimmte Verhaltensmuster aufgezeigt werden, um eine Diagnose zu bestätigen oder einen individuellen Therapieplan zu entwickeln [12]. Intraindividuelle Veränderungen im Zeitverlauf können durch digitales Phänotypisieren gut aufgedeckt werden [29].

Die Forschung zu digitalem Phänotypisieren für die psychische Gesundheit hat großes Potenzial, steht jedoch noch ganz am Anfang. Beispielsweise muss untersucht werden, welche digitalen Biomarker für welche Art der Erkrankung den meisten Informationswert haben und wie sie die Regelversorgung und den Verlauf einer Psychotherapie oder digitalen Intervention optimieren können [32]. Auch ethische Fragen werden aufgeworfen und es muss geklärt werden, wem die Daten eines/r Smartphonebesitzers/Smartphonebesitzerin gehören und wie mit deren Vertraulichkeit umgegangen wird [33]. Transparenz in Bezug auf die Sammlung der Daten muss gegeben sein und der/die Patient:in muss darüber unterrichtet werden, welche Daten gesammelt werden (bspw. Text- oder Sprachnachrichten, E‑Mails, GPS-Daten), mit welchen Geräten (bspw. Smartphone, Laptop) und zu welchen Zeitpunkten [34]. Die Einwilligungserklärung für eine etwaige Studienteilnahme sollte diese Informationen beinhalten wie auch eine Offenlegung, wer Zugang zu den gesammelten Rohdaten des/der Patienten:in hat und wie diese anonymisiert werden. Wissenschaftler:innen haben auch dazu aufgerufen, Patient:innen über Schlussfolgerungen, die aus den Daten gezogen werden können, aufzuklären [35]. Eine mögliche Patientenaufklärung, wie bspw. durch ein Informationsblatt, sollte auch Grenzen der potenziellen Datenanalyse aufzeigen und mögliche kausale Inferenzen ansprechen [34].

## Chancen und Herausforderungen bei digitalen Interventionen im Bereich der psychischen Gesundheit

Gegenüber traditioneller Psychotherapie haben digitale Technologien für die psychische Gesundheit einige Vorteile. Diese umfassen beispielsweise den leichteren Zugang zur Hilfe oder Selbsthilfe. Zudem können digitale Interventionen weniger stigmatisierend für Patient:innen sein, da die Teilnahme anonym sein kann. Vorteilhaft ist auch, dass digitale Interventionen personalisierbar sind, also an die/den Endnutzer:in angepasst werden können. Durch digitale Phänotypisierung ergeben sich zudem neue Möglichkeiten in der Psychotherapieforschung. Durch Programmierung und die weitere Zugabe von Daten (bspw. Daten über den Schlaf) könnte eine digitale Intervention auf die individuellen Bedürfnisse der jeweiligen Nutzenden zugeschnitten werden [36]. Zudem könnten hybride Formen aus traditioneller Psychotherapie und digitalen Interventionen zum Beispiel bei der Diagnosestellung angewendet werden, um das Gesundheitssystem zu entlasten und knappe Ressourcen zu schonen.

Schätzungen zufolge gibt es mehr als 10.000 digitale Interventionsformen für die psychische Gesundheit, die zum Download in App-Stores bereitstehen [37]. Allerdings sind nur ca. 2 % dieser zum Download bereitstehenden Interventionen, die auf kommerziellem Weg erhältlich sind, klinisch getestet und evidenzbasiert [38, 39]. Manche dieser kommerziellen digitalen Interventionen können der allgemeinen Bevölkerung zur Entspannung oder psychologischen Selbsthilfe dienlich sein. Bei Menschen, die unter einer schweren Symptomatik leiden, sollte allerdings sichergestellt werden, dass die digitale Intervention den individuellen Bedürfnissen wirklich gerecht wird und es zu keiner Verschlechterung der Symptomatik kommt.

Eine ärztliche Verordnung von digitalen Interventionen wird in Deutschland durch das Digitale-Versorgung-Gesetz (DVG) geregelt, welches im Dezember 2019 in Kraft getreten ist [40]. Digitale Gesundheitsanwendungen müssen, um verordnet werden zu können, durch das Bundesministerium für Arzneimittel und Medizinprodukte (BfArM) zugelassen werden und werden umfassend geprüft. Um zugelassen zu werden, muss eine neue digitale Gesundheitsanwendung eine patientenrelevante Struktur- und Verfahrensverbesserung in der Versorgung erzielen [41] sowie eine positive Kosten-Nutzen-Bilanz aufzeigen, welche vorzugsweise durch eine randomisierte kontrollierte Studie belegt wurde [42]. Aktuell (Januar, 2024) gibt es 49 zugelassene digitale Gesundheitsanwendungen in Deutschland, wobei 24 für psychische Erkrankungen verfügbar sind [43]. Wie bei allen Verordnungen sollte auf Präferenzen von Patient:innen eingegangen werden; beispielweise wünschen sich nicht alle Patient:innen eine digitale Intervention und ziehen ein Beratungsangebot in traditioneller Form mit einem Psychologen oder einer Psychologin vor [44].

Digitale Gesundheitsanwendungen können positive Auswirkungen auf eine psychische Symptomatik haben, es ist jedoch zu bedenken, dass digitale Technologien für die psychische Gesundheit soziale Ungleichheiten in der Gesellschaft weiter verfestigen können, wenn sie z. B. nur von Personen mit hohen technischen oder sozialen Ressourcen genutzt werden. Es ist deshalb wichtig, Patient:innen, aber auch Therapeut:innen in der digitalen Intervention zu schulen, um deren „digitale Gesundheitskompetenz“ (Digital Health Literacy) und Verständnis für den Ansatz zu erhöhen. Es hat sich zudem gezeigt, dass eine nicht gut angepasste digitale Intervention (die beispielweise nicht zu den kulturellen Werten und Ansichten einer Person passt) häufig nicht bis zum Ende geführt wird [22]. Deshalb erscheint eine Adaption an die Sprache und die kulturellen Werte der Endnutzer:innen besonders wichtig. Beispielsweise zeigen aktuelle Studien, dass Patient:innen mit Demenz, ihre pflegenden Angehörigen und Pflegefachkräfte bisher noch unzureichend in die Entwicklung von digitalen Technologien einbezogen werden [45].

Zwei der größten Herausforderungen sind die langfristige Integration digitaler Interventionen in den Behandlungsalltag sowie das Engagement und die kontinuierliche Teilnahme von Patient:tinnen. Studien zeigen, dass einige digitale Interventionen an Effektstärke verlieren, sobald sie in den Behandlungsalltag (außerhalb streng kontrollierter Studien) implementiert werden. Dieses Phänomen ist in Implementationsstudien häufiger zu beobachten, wenn eine kontextspezifische Implementationsstrategie fehlt [46]. Beispielsweise zeigte eine der größten Implementationsstudien, die zwei webbasierte Interventionen für Depression mit einer Standardbehandlung für Depression im nationalen Gesundheitssystem des Vereinigten Königreichs verglichen hat, keinen Effekt gegenüber der Standardbehandlung [47].

## Arbeiten zu Public Mental Health im Leibniz WissenschaftsCampus Digital Public Health (LWC DiPH)

### Weiterentwicklung einer psychologischen Kurzintervention

Der LWC DiPH betreibt im Bereich der psychischen Gesundheit wichtige nationale und internationale Forschung. Eines der Projekte beschäftigt sich mit der digitalen Weiterentwicklung der transdiagnostischen Kurzintervention „Problem Management Plus“ (PM+), die von der Weltgesundheitsorganisation (WHO) empfohlen wird und speziell für Populationen geeignet ist, die psychologischen Belastungen und widrigen Umständen ausgesetzt waren (bspw. Geflüchtete; Populationen mit Gewalterfahrungen o. Ä.; [48]). PM+ kann im Einzel- oder Gruppenformat durchgeführt werden. Vorgesehen sind 6 Sitzungen, die nicht von Psychotherapeut:innen, sondern von Laien (bspw. Sozialarbeiter:innen, freiwilligen Helfer:innen) durchgeführt werden.

PM+ hat sich als effektive und kostenwirksame Intervention etabliert. Sie wurde in mehreren randomisierten Kontrollstudien getestet, darunter auch in Pakistan [49]. PM+ wird in Pakistan von Gemeindehelferinnen, sogenannten Lady Health Workers angeboten. Um die Verbreitung der Intervention in Pakistan zu beschleunigen, hat der LWC DiPH gemeinsam mit Partnereinrichtungen in Pakistan begonnen, PM+ in digital unterstützter Form anzubieten (sogenanntes Technology-Assisted Problem Management Plus, TA-PM+). TA-PM+ ist eine App, die den Gemeindehelferinnen auf einem Tablet zur Verfügung gestellt wird und durch die PM+-Anwendung und die einzelnen Sitzungen führt. Jede Sitzung wird begleitet von an die lokale Situation angepassten Kurzfilmen, die den Patient:innen einzelne Strategien (bspw. Atemübungen) näherbringen. Dies geschieht mithilfe von Avataren („Dr. Ayesha“, „Gemeindehelferin Shabana“ und „Patientin Shazia“), die den Inhalt der einzelnen Sitzungen im Kurzvideo veranschaulichen. TA-PM+ wurde zusammen mit der lokalen Bevölkerung in Pakistan entwickelt und in mehreren Durchläufen an die Bedürfnisse der Gemeindehelferinnen und der Patient:innen angepasst. Momentan wird TA-PM+ in einer Machbarkeitsstudie in Pakistan getestet. Eine umfassende Wirksamkeitsstudie zu Effektivität und Implementierung plant der LWC DiPH je nach Verlauf im Anschluss.

### Technologie für Menschen mit Demenz

Unter dem Oberbegriff „Demenz“ werden mehr als 100 neurokognitive Erkrankungen gefasst, die die Funktion des Gehirns und insbesondere Gedächtnis, Verhalten, Kognition und soziale Fähigkeiten stark beeinträchtigen [50]. Demenz verringert in erheblichem Maß die Fähigkeit eines Menschen, alltägliche Aktivitäten auszuführen [51], und ist weltweit eine der Hauptursachen für Pflegebedürftigkeit bei älteren Erwachsenen [52]. Eine kurative Therapie demenzieller Erkrankungen gibt es derzeit nicht, sodass sie insbesondere in den Fokus einer unterstützenden Versorgung mit dem Blick auf Autonomie, soziale Teilhabe und Beziehungspflege rücken [53]. Hierbei werden zunehmend auch technologische Lösungen als Strategien zur Optimierung bestehender Versorgungsstrukturen in den Blick genommen [54]. Allerdings ist derzeit nur wenig darüber bekannt, ob diese Technologien die soziale Teilhabe und die Aufrechterhaltung von Beziehungen bei Menschen mit Demenz, die mit ihren versorgenden Angehörigen noch in der eigenen Häuslichkeit leben, unterstützen können. Um diese Wissenslücke zu schließen, wurde im Rahmen des LWC DiPH eine Machbarkeitsstudie unterstützt, die die Auswirkungen des Einsatzes des technologiegestützten Aktivierungssystems „I-CARE“, das speziell für Menschen mit Demenz und ihre versorgenden Angehörigen entwickelt wurde, evaluiert hat [55].

Bei der getesteten Technologie handelt es sich um ein Tablet-basiertes Aktivierungssystem, das ein breites Spektrum an Aktivitäten, darunter Bildergalerien, Videos, Kurzgeschichten, Sprichwörter, Quizze und Spiele umfasst, die Menschen mit Demenz zusammen mit ihren versorgenden Angehörigen durchführen können [56]. Die Aktivitäten sind dabei unterschiedlich komplex und verschiedenen Schwierigkeitsstufen zugeordnet, sodass das System für die meisten Altersgruppen, Arten und Stadien der Demenz geeignet ist. Zudem ist über eine Feedback-Funktion die Anpassung von Inhalten an die Präferenzen der Nutzer:innen möglich. Zu Beginn jeder von I‑CARE unterstützten Sitzung fragt das System nach dem täglichen Wohlbefinden des Menschen mit Demenz und schlägt vier Aktivitäten vor, die auf persönlichen Informationen wie Alter, früherer Beruf und Interessen sowie auf Bewertungen aus früheren Sitzungen basieren. Versorgende Angehörige können Inhalte auch über die Suchfunktion des Systems oder die Aktivitätshistorie auswählen.

Im Rahmen einer Mixed-Methods-Studie mit Prä‑/Posttest-Design und Follow-up wurden von Dezember 2020 bis Dezember 2021 versorgende Angehörige von Menschen mit Demenz über lokale Gesundheits- und Pflegeorganisationen, Selbsthilfegruppen und Informationszentren in und um Bremen rekrutiert, um I‑CARE zu evaluieren [55].

Die Ergebnisse dieser Studie deuten darauf hin, dass I‑CARE ein praktikables Instrument ist, um positive Erfahrungen zwischen Menschen mit Demenz und ihren versorgenden Angehörigen zu ermöglichen. Wichtige Beziehungsergebnisse für die teilnehmenden Dyaden waren die Bereicherung sozialer Interaktionen, die Erleichterung der Kommunikation, die Durchführung gemeinsamer Aktivitäten und die Aufrechterhaltung der Beziehung. Das System wurde von den meisten Teilnehmer:innen als benutzerfreundlich empfunden. Neben technischen Weiterentwicklungen von I‑CARE sollte für eine breite zukünftige Nutzung auch darauf geachtet werden, dass Nutzer:innen eine kontinuierliche proaktive Unterstützung erfahren, die auf ihre Bedürfnisse und Voraussetzungen zugeschnitten ist, um das System selbstständig und konstruktiv nutzen zu können.

## Diskussion

Dieser Beitrag beleuchtete digitale Technologien für die psychische Gesundheit und zeigte deren Chancen und Grenzen auf. Exemplarische Arbeiten aus dem Leibniz WissenschaftsCampus Digital Public Health wurden illustriert. Es gibt diverse Möglichkeiten, die psychische Gesundheit mittels neuer digitaler Technologien zu unterstützen oder zu verbessern. Apps werden mittlerweile häufig angewandt, diese können als alleinige Interventionsform benutzt werden oder im hybriden System traditionelle Psychotherapie unterstützen. Zudem können digitale Interventionen dem/der Therapeut:in dabei helfen, eine Beratung oder Behandlung durchzuführen. Beispiele hierzu sind neue Innovationen wie TA-PM+ und I‑CARE aus dem LWC DiPH. Wichtig ist, dass digitale Technologien evidenzbasiert sind. Für Depression und Angststörungen zeigt sich eine gute Datenlage in Bezug auf Apps, die dem Ansatz der kognitiven Verhaltenstherapie folgen, und Effekte sind vielversprechend.

In Bezug auf die Demenz gibt es Forderungen zu umfassenderen Studien zu Akzeptanz, Effektivität und Effizienz von digitalen Technologien für Menschen mit Demenz und ihre versorgenden Angehörigen. Ergänzend braucht es eine stärkere Diskussion, wie aktuelle und künftige Technologien zur Förderung der sozialen Teilhabe bei Demenz eingesetzt und genutzt werden können und sollen. Die klinische Praxis könnte auch von der Erstellung und Nutzung von Leitlinien profitieren, wenn es darum geht, soziale Technologien in der Versorgung und Pflege von Menschen mit Demenz einzusetzen. Hier sei beispielsweise auf den Leitfaden „Best Practice Guidance: Human Interaction with Technology in Dementia“ verwiesen, der im Rahmen der EU-geförderten „Marie Skłodowska-Curie Innovative Training Networks“ (MSC-ITN), INDUCT (2016–2020) und DISTINCT (2019–2023), entwickelt wurde. Dieser enthält umfangreiche Empfehlungen zur Verbesserung von Technologien zur Unterstützung von Menschen mit Demenz in den Bereichen Alltagsleben, sinnvolle Aktivitäten und Gesundheitsversorgung [57].

Für den Behandlungsalltag von Menschen mit psychischen Erkrankungen wie Depression, Angststörungen oder Demenz gibt es einige Implementationshürden zu überwinden, welche eine langfristige Integration einer digitalen Intervention in das Gesundheitssystem behindern können. Die Gründe hierfür sind vielschichtig. Beispielweise impliziert eine langfristige Integration digitaler Innovationen für die psychische Gesundheit meist eine Veränderung prozeduraler Abläufe im Behandlungsalltag. Dies kann je nach Implementationsstandort und Ressourcenlage (finanzielle Ressourcen und Menge an Personal) auch eine neue Definition von Rollen und Verantwortlichkeiten mit sich bringen oder eine Veränderung der Personalstruktur bedingen. Diskutiert wird die Einführung sogenannter digitaler Navigatoren für die psychische Gesundheit [8], die dafür verantwortlich sind, Patient:innen in digitalen Interventionen zu schulen, bei technischen Problemen weiterhelfen und die Patient:innen motivieren, die Intervention vollständig zu durchlaufen. Insgesamt beinhaltet die Einführung einer neuen Intervention, auch in digitaler und hybrider Form, eine Schulung des beteiligten Personals. Die Forschung bestätigt die Wichtigkeit dieser Schulungen, da Einstellung und Wissen von Therapeut:innen zentral sind und einen wesentlichen Einfluss auf den Nutzen und die Teilnahme der Patient:innen an der digitalen Intervention haben [12, 58].

Eine der größten Schwierigkeiten ist es, Patient:innen in der digitalen Intervention zu halten und unvollständige Interventionsdurchführung zu minimieren. Entsprechende Strategien wurden erarbeitet [24]. Dabei zeigt sich, dass geleitete digitale Interventionen die Teilnahme erhöhen können und wahrscheinlich auch einen größeren Effekt haben als ungeleitete [25, 59]. Trotzdem besteht in Hinblick auf individuelle Differenzen bei der Teilnahme an digitalen Interventionen für die psychische Gesundheit noch Forschungsbedarf. Soziodemografische Charakteristika wie Alter, Geschlecht und Herkunft können die Teilnahme an digitalen Interventionen beeinflussen [59] und sich beispielsweise negativ auf die Interaktion mit Chatbots auswirken [38]. Intraindividuelle Differenzen scheinen hier auch eine Rolle zu spielen. Beispielsweise hat es sich gezeigt, dass Menschen mit höherer Selbstwirksamkeit eher eine digitale Intervention zu Ende führen als Menschen, die nicht auf sich vertrauen und glauben, dass sie ihre psychische Gesundheit nicht selbst steuern können [38]. Zusätzlich muss das Vertrauen von Patient:innen in die digitale Intervention gestärkt werden. Beispielsweise müssen Patient:innen darauf vertrauen können, dass Daten nicht an Dritte weitergegeben werden und Datenschutzrichtlinien eingehalten werden.

Hybride Formen, also die Verknüpfung von traditioneller Verhaltenstherapie mit digitalen Interventionen, erscheinen sinnvoll. Sie sind gerade auch in Bezug auf die therapeutische Allianz wichtig, da die Beziehung zwischen Therapeut:in und Patient:in einen klaren Einfluss auf die Wirksamkeit der Therapie hat [60]. Bei digitalen Interventionen, die als alleinige Interventionsform angewendet werden oder ungeleitet sind, könnte der Zuwert der „therapeutischen Allianz“ verloren gehen. Ob sich bei geleiteten digitalen Interventionen die Allianz zwischen Patient:in und Therapeut:in genauso gut herstellen lässt wie in der traditionellen Form der Psychotherapie, muss noch weiter erforscht werden. Auch kann die alleinige Nutzung von Chatbots zur Diagnosestellung bei psychiatrischen Erkrankungen zu Fehleinschätzungen führen. Für Diagnosen verwenden Chatbots häufig spezifische symptombasierte Ein- und Ausschlusskriterien, die von einem/einer Therapeuten:in nochmals überprüft werden sollten. Dies ist besonders wichtig, wenn Patient:innen Komorbiditäten zu einer psychischen Erkrankung aufweisen.

## Fazit

Digitale Interventionen für die psychische Gesundheit können bei verschiedenen Krankheitsbildern Diagnose und Therapie unterstützen. Das Potenzial ist groß, allerdings sind digitale Interventionen nicht mit der persönlichen Behandlung durch Therapeut:innen gleichzusetzen. Hybride Formen erscheinen vielversprechend. In der Zukunft muss jedoch weiter erforscht werden, wann, wie und für wen digitale Interventionen für die psychische Gesundheit von Vorteil sind und wie diese im Behandlungsalltag langfristig integriert werden können.
